# New Perspectives Provided by Merging Computed Tomographic Scanning and Electroanatomical Mapping of Koch’s Pyramid

**DOI:** 10.3390/jcdd13040168

**Published:** 2026-04-14

**Authors:** Claudio Pandozi, Andrea Matteucci, Robert H. Anderson, Marco Galeazzi, Maurizio Russo, Gianmaria De Filippis, Massimiliano Danti, Marco Valerio Mariani, Carlo Lavalle, Andrea Bassi, Maurizio Malacrida, Mauro Bura, Furio Colivicchi

**Affiliations:** 1 Clinical and Rehabilitation Cardiology Division, San Filippo Neri Hospital, 00135 Rome, Italy; 2 Biosciences Institute, Newcastle University, Newcastle-upon-Tyne NE2 4HH, UK; 3 COU Diagnostic Imaging, M.G. Vannini Hospital, 00177 Rome, Italy; 4 Department of Cardiovascular, Respiratory, Nephrological, Anesthesiological and Geriatric Sciences, “Sapienza” University of Rome, 00189 Rome, Italy; 5 Santa Lucia Foundation, IRCCS, 00179 Rome, Italy; 6 Boston Scientific, 20134 Milan, Italy

**Keywords:** AV nodal re-entrant tachycardia, Koch’s triangle, nodal potential, computed tomography, imaging integration

## Abstract

Background: Atrioventricular (AV) nodal re-entrant tachycardia (AVNRT) is strongly related to the anatomy and physiology of the AV nodal and junctional area. Objectives: This study aims to precisely ascertain the localization of structures within Koch’s triangle by employing the recording of nodal potentials in conjunction with the integration of three-dimensional electrical maps merged with computed tomographic images. Methods: Five consecutive patients with typical AVNRT and an available cardiac computed tomographic scan were enrolled. High-resolution mapping was performed prior to the initial ablation attempt. Results: The low-frequency, low-amplitude humped nodal potential was consistently detected within the presumed compact node location, found in the superior septal area in three patients and in the mid-paraseptal region in two cases. The length of the region was 4.5 ± 1.2 mm, with its width measured at 2.7 ± 0.6 mm (distance from the atrioventricular membranous septum = 3 ± 0.8 mm). The nodal potential was consistently recorded alongside the slow pathway potential in the infero-septal region, anterior to the orifice of the coronary sinus (distance from the slow pathway potential to the site of His potential = 15 ± 3.2 mm). This suggests that the slow pathway electrogram likely represented the medial or distal portion of the inferior nodal extension, rather than the node itself. In all patients, successful ablation was achieved, requiring a median of 5 [4–6] radiofrequency deliveries. No procedural complications were encountered. Conclusions: This study, which integrates three-dimensional electroanatomical maps with reconstructed computed tomographic datasets and utilizes specific anatomical landmarks, provides a reliable and accurate estimation of the atrioventricular conduction axis components in relation to the Koch’s pyramid boundaries.

## 1. Introduction

Over a century has elapsed since the German pathologist Robert Koch [1] first described the triangular anatomical region, now bearing his name, which hosts the cardiac conduction system. To this day, the area continues to harbor unresolved mysteries. Indeed, a complete understanding of the electroanatomical mechanisms governing the activation of its walls during both sinus rhythm and re-entrant arrhythmias remains elusive. The triangle is bounded by the Eustachian ridge, containing the tendon of Todaro, and the hinge line of the septal leaflet of the tricuspid valve, which comes together at its apex. Its base is formed by the orifice of the coronary sinus and the septal isthmus [2]. Its wall is separated from the crest of the ventricular septum by the fibro-adipose tissue filling the inferior pyramidal space [3]. Its apex is adjacent to the inferoseptal recess of the subaortic outflow tract, the two separated by the atrioventricular component of the membranous septum. Therefore, a more realistic 3D description of the conduction system area has been made and thus the two-dimensional concept of the Koch triangle has been replaced by the three-dimensional description of the Koch pyramid according to its anatomical relationship to the pyramidal space and the infero-septal recess [3]. Defining the complex boundaries of Koch’s triangle and pyramid remains challenging for electrophysiologists. Even the use of three-dimensional mapping systems to delineate the geometry can introduce potential pitfalls. For example, the use of electrocardiographic criteria, such as the loss of the atrial electrogram and the presence of a large ventricular electrogram, to pinpoint the insertion of the septal leaflet of the tricuspid valve can lead to errors because the septal leaflet is apically displaced, resulting in a atrio-ventricular septal or paraseptal area where a high amplitude ventricular electrogram can be recorded. Furthermore, until very recently, it was not possible to define electrically the location of the atrioventricular node, despite promising initial attempts to define a “nodal potential” [4]. Consequently, previous studies have concentrated on the propagation of the atrial inputs to the node [5]. Recently, nonetheless, using a mapping system in conjunction with a small-sized electrode basket catheter, we have identified potentials originating not only from the compact node but also its inferior extensions [6,7]. Despite recent advances, at present it remains impossible directly to visualize the atrioventricular conduction axis when using standard imaging techniques [8]. Predictions regarding its location can be made based, nonetheless, using known anatomical landmarks coupled with documented dimensions of the components of the axis and their distances from the landmarks. The components of the conduction axis, however, exhibit anatomical variability [9]. Confirmation of its location, therefore, requires the recording of electrograms specific to the identified anatomical structures on the electro-anatomic map. Taking all these features into consideration, we propose that the recording of nodal potentials, in combination with the integration of three-dimensional electro-anatomic maps merged with computed tomographic images, can delineate the location of the structures within the region of Koch’s triangle. Such an approach, if successful, can ultimately enhance the effectiveness and safety of ablation procedures in the septal, paraseptal, and/or parahisian regions. The aim of this study is to provide a proof of concept that the integration of nodal potential recordings with CT-based anatomical reconstruction can improve the localization of conduction system components within Koch’s pyramid.

## 2. Materials and Methods

From March 2021 to July 2022, a total of 5 consecutive patients with symptomatic supraventricular tachycardia, a suspected clinical diagnosis of typical atrioventricular nodal re-entry tachycardia, and an available cardiac computed tomographic scan, performed for the appearance during tachycardia of chest pain, ventricular repolarisation abnormalities and/or troponin elevation, were enrolled and underwent high-resolution mapping before the first attempt at ablation. The included patients were part of the CHARISMA (ClinicalTrials.gov Identifier: NCT03793998), a multi-center, prospective, single-arm cohort study aimed at describing the Italian clinical approach to ablation of various types of arrhythmias using a high-density mapping system in real-world practice. The study was conducted in accordance with the Declaration of Helsinki. Ethical review and approval were waived due to the observational nature of the study and because all procedures were part of routine clinical practice with no experimental interventions.

### 2.1. Electrophysiological Study and Mapping Before Ablation

A steerable decapolar catheter was introduced into the coronary sinus via the right or left femoral veins, while standard quadripolar catheters were placed under fluoroscopy in the high right atrium, right ventricle, and the His bundle area. To investigate the presence of dual nodal pathways, we employed atrial programmed stimulation and examined the Kay-Epstein sign during incremental atrial pacing [10]. Tachycardia was successfully induced through atrial programmed extrastimulus testing, and the diagnosis of nodal reentry was firmly established following standard criteria and diagnostic pacing maneuvers as described elsewhere [11]. To facilitate a comprehensive understanding of atrial activation during both sinus rhythm and nodal tachycardia, we harnessed the power of a basket catheter, which was equipped with 64 electrodes, each covering an area of 0.4 mm^2^, spaced 2.5 mm apart (Orion, Boston Scientific, Natick, MA, USA), and the Rhythmia EA mapping system (Rhythmia HDx SW version 4.5, Boston Scientific). Previous research by Scherlag et al. in the ex vivo Langnedorff perfused dog heart [4] had successfully obtained transmembrane action potentials, allowing them to correlate nodal cell activations with bipolar extra-cellular recordings in the region of the triangle. Moreover, histological samples from below the electrodes recording the presumed AV nodal bipolar potential showed the presence of tissue characteristic of the AV node. They also elucidated the behavior of nodal electrograms in both sinus rhythm and slow-fast tachycardia using electrophysiological maneuvers [4,12]. Notably, Scherlag et al. demonstrated that the initiation of action potentials in the compact node and node-His transition zone coincided with the middle and end of the extracellular potentials recorded from the axis, respectively [4,12].

**Figure 1:**
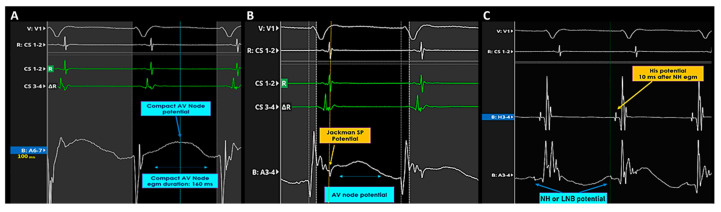
Recordings of AV node potential in Koch’s triangle during AVNRT. (**A**) In the mid-septal region of Koch’s triangle, a long-duration, low-frequency, low-amplitude AV node potential not preceded by a Jackmann slow pathway potential, was recorded without any near-field or far-field His electrogram. The potential was annotated in the middle of its duration. (**B**) Recording of AV node potential in the slow pathway region of Koch’s triangle during AVNRT. Left side: in the infero para-septal region of Koch’s triangle, a long-duration, low-frequency, low-amplitude AV node potential was recorded together with a Jackmann slow pathway potential slightly before its onset. The potential was annotated at the peak of the Jackmann slow pathway potential. (**C**) Electrogram recorded in the supero-septal region of Koch’s triangle during AVNRT: the low-frequency His-like potential was presumed to represent the compact node-His transition zone potential or the lower nodal bundle electrogram.

**Figure 2:**
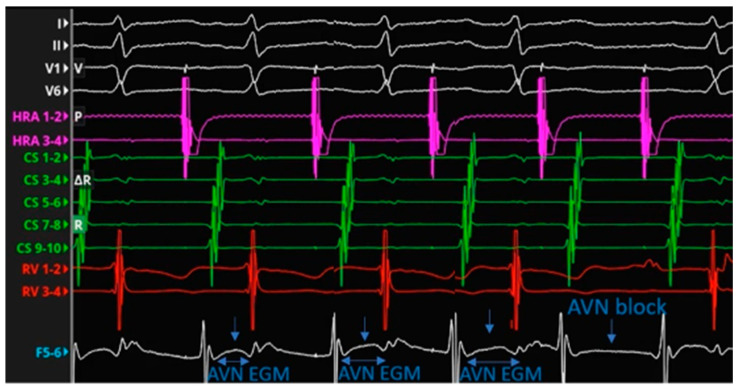
AV node potential recorded by spline F 5-6 during a Wenckebach cycle induced by incremental atrial pacing. The compact AV node potential completely disappears after the atrial non-conducted beat.

Electrical mapping was performed using the Orion 64-electrode basket catheter (electrode area 0.4 mm^2^; interelectrode spacing 2.5 mm) and the Rhythmia HDx mapping system (software version 4.5, Boston Scientific) during sinus rhythm and, when sustained, during AVNRT. Bipolar electrograms were analyzed using a non-conventional filter setting of 0.05–250 Hz to preserve low-frequency, low-amplitude components potentially arising from AV nodal structures. Candidate compact AV nodal potentials were defined as long-duration, low-frequency, low-amplitude humped signals located between the atrial and ventricular electrograms, not preceded by a Jackman slow-pathway potential and recorded in the absence of a near-field or far-field His electrogram. In these cases, the potential was annotated at the midpoint of its duration. When a low-frequency His-like component followed the humped potential, the electrogram was annotated at its end, consistent with the presumed node-His transition or lower nodal bundle region. Jackman slow-pathway potentials were annotated at their peak. When a slow-pathway potential was followed by a humped low-frequency component in the infero-paraseptal region, the latter was interpreted as compatible with activation of the right inferior nodal extension (Figure 1). To distinguish slow-pathway activity from ventricular electrogram components during tachycardia, His-refractory ventricular extrastimulus testing was performed. In addition, incremental atrial pacing, observation of Wenckebach sequences, and adenosine administration were used as adjunctive maneuvers to assess rate-dependent changes and disappearance of putative nodal potentials (Figure 2).

### 2.2. Patient Preparation for CT Scan

Patients with a heart rate exceeding 65 beats per minute, unless contraindicated, received a single intravenous dose of 5 mL of metoprolol tartrate (Seloken, Innova Pharma, London, UK) 45 min before the study. Sublingual isosorbide dinitrate (Carvasin 5 mg, Teofarma, Valle Salimbene, Italy) was administered shortly before the computed tomographic scan.

### 2.3. Scan Protocol and Image Reconstruction

We used a 64-slice scanner (Brillance CT 64-channel, Philips, Best, The Netherlands). An initial scan without contrast was used precisely to define the start and end points of the volume to be included in the acquisition and to calculate the calcium score (SmartScore protocol). The acquisition package included the ascending aorta superiorly, approximately 1 cm above the tracheal bifurcation, and the lower portion of the lowest hemidiaphragm. This permitted examination of the entire cardiac volume, with a perfect visualization of the four cardiac chambers, the planes of the valves, and the pulmonary veins. During this pre-contrast phase, the main scanning parameters were as follows: beam collimation of 40 × 0.625 mm, slice thickness of 2.5 mm, table translation of 1 cm every 4 acquisitions of 2.5 mm, tube rotation speed of 0.4 s, tube voltage of 120 kV, radiation beam intensity of 75 mA, field of view (FOV) of 25 cm, and craniocaudal scan direction. Retrospective cardiac gating was used for cardiac synchronization. A second scan was then taken after the intravenous injection of organo-iodinated contrast medium, using an automatic dual-head injector (Stellant, MEDRAD, Pittsburgh, PA, USA), through an 18-gauge cannula placed in an antecubital vein in the arm. 80 mL of non-ionic organo-iodinated contrast medium (Iomeron 400, Bracco, Milan, Italy) was administered, followed by 40 mL of physiological saline infusion at a flow rate of 5 mL/s. The bolus tracking technique was used to synchronize the arrival of the contrast medium in the coronary arteries with the start of the acquisition. During the contrast phase, the main scanning parameters were as follows: beam collimation of 64 × 0.625 mm, layer thickness of 0.9 mm, reconstruction increment of 0.625 mm, table advancement speed of 2.9 mm/rotation, tube rotation speed of 0.35 s, tube voltage of 120 kV, radiation beam intensity between 400 and 800 mA, FOV of 25 cm, and craniocaudal scanning direction. The scanning time during the contrast phase was 9 s. Image reconstruction was performed using three fixed temporal windows set at 70%, 75%, and 80% of the cardiac cycle, the period between two consecutive R-R waves of the electrocardiographic trace, corresponding to the meso- to telediastolic phase. In cases of motion artifacts due to an increase or sudden change in heart rate during the acquisition, we used other reconstruction windows, such as from 40% to 65% of the R-R cycle.

### 2.4. Merged Acquisition

**Figure 3:**
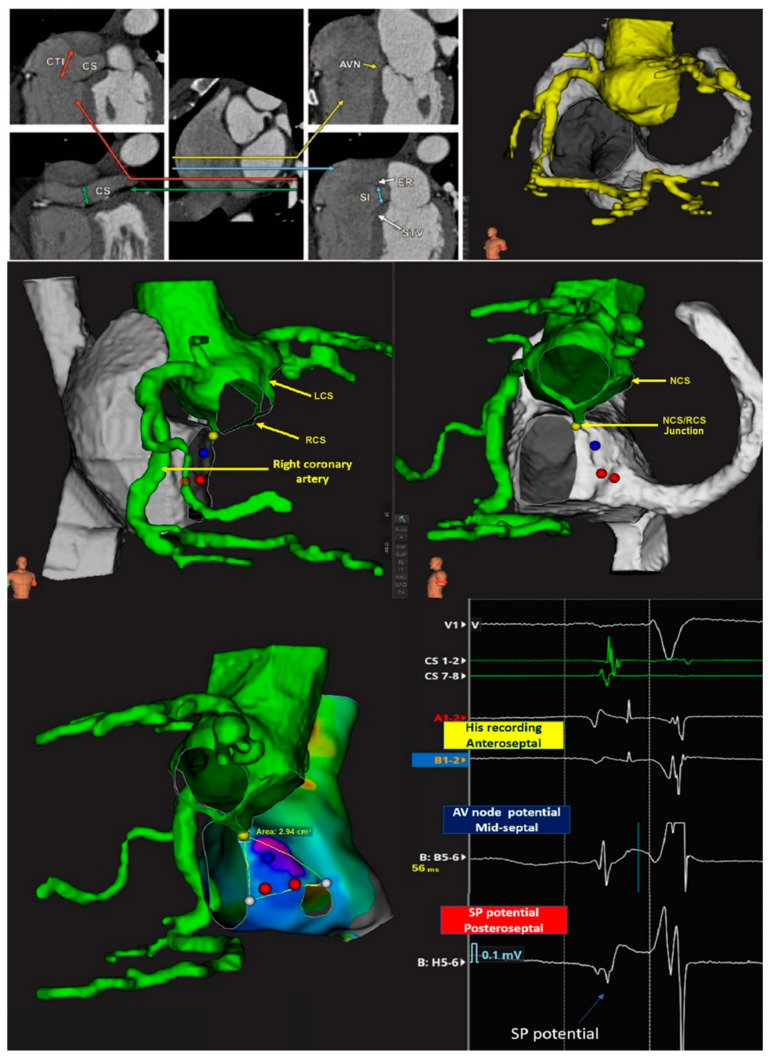
Merged acquisition from 3D mapping and reconstruction of computed tomographic datasets: step per reconstruction. Once the RA Rhythmia™ map has been acquired using Orion catheter, we aligned it with the reconstructed segmentations. This alignment allows us to clearly identify the aortic root relative to Koch’s pyramid. Lower panels: The position of the non-branching atrioventricular bundle inside the His cloud was identified at the site where the infero-septal recess of the aortic root meets the apex of the pyramid of Koch. The inferior right panel shows the electrograms recorded in Koch’s triangle. CTI = cavo-tricuspid isthmus, CS = coronary sinus, AVN = atrioventricular node, LCS = left coronary sinus, RCS = right coronary sinus, NCS = non-coronary sinus.

We employed Geometry Import to bring in geometries generated through the tomographic scans and align them with the previously established anatomical map made using the Rhythmia^TM^ Mapping System, thus facilitating comparative analysis of the imported geometry. An import tool was used to import pre-acquired tomographic images in DICOM format, and ITK-SNAP software (https://www.itksnap.org/pmwiki/pmwiki.php) was employed for highly reliable and effective segmentation of the cardiac chambers [13]. The ITK-SNAP software permitted is to segmentation of the aorta and coronary arteries (along with the right atrium and the coronary sinus. After acquiring the RA Rhythmia^TM^ map using the Orion catheter, we aligned it with the segmented tomographic images. This permitted us clearly to discern the position of the aortic root relative to Koch’s triangle (Figure 3).

We also reconstructed the aortic valvar leaflets precisely to locate the near-field His recording within the His cloud on the right side along the atrioventricular membranous septum, corresponding to the site of the inter-leaflet triangle between the right and non-coronary aortic sinuses on the left side and identified the hinge of the septal leaflet of the tricuspid valve. We then used the location of the right coronary artery, as reconstructed from the tomographic images to locate the other leaflets of the tricuspid valve. The precise location of Todaro’s tendon, however, could not be determined. We defined the posterior boundary of the triangle, therefore, as a closed line segment, bordered on the left side by the membranous septum, and on the right side by the point where the basal edge contacts the Eustachian valve and ridge [5,14]. Once the merging process was completed, we confirmed the accuracy of the merge, particularly the position of the valvar leaflets, by displaying the location of a mapping catheter simultaneously on the intracardiac echo and the merged map.

## 3. Results

**Figure 4:**
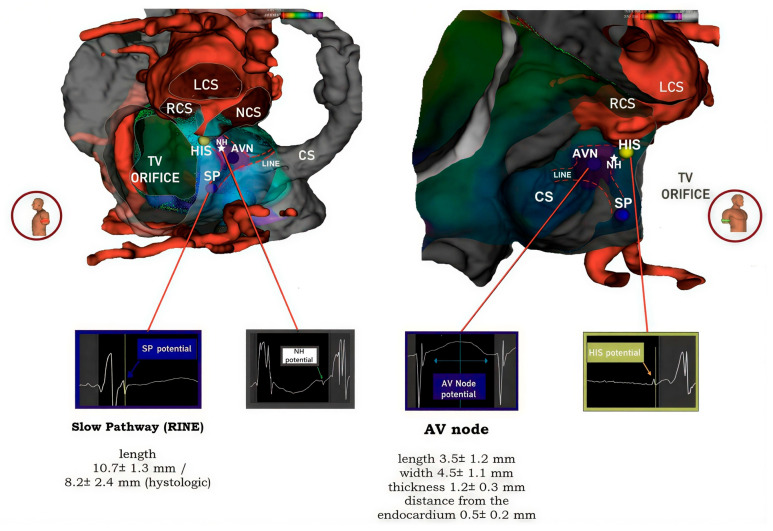
Merging of the electroanatomical mapping with the reconstructed computed tomographic dataset. As shown in the figures, the electroanatomical map was overlaid with scans obtained before the procedure, resulting in a combined image with anatomical markers of the boundaries of the pyramid of Koch. **Left**: The left anterior oblique view shows, with an external view, the location of the His bundle recording site, the transition between His and the AV node (NH) and the area of the compact AV node, continuing along the tricuspid annulus with the right inferior nodal extension, identified on the basis of the Jackmann slow pathway potential preceding a low frequency humped potential. The conduction axis is directed towards the infero-septal recess of the left ventricle at the apex of Koch’s pyramid [3]. **Right**: The right anterior oblique view shows the view of the septal surface of Koch’s triangle area as seen from the right atrium. The electrograms recorded in these areas using the settings described in the text are shown below. The specified measurements of the atrial components of the conduction axis, including the depth to endocardial surface and the location of the slow pathway, has initially been taken from the histological studies described in references [14,15], but the actual position of the conduction system structures were confirmed by the recording of their specific electrograms. CS = coronary sinus, AVN = atrioventricular node, LCS = left coronary sinus, RCS = right coronary sinus, NCS = non-coronary sinus, SP = slow pathway or right inferior nodal extension, LINE = left inferior nodal extension, TV orifice = tricuspid valve orifice.

After merging the map with the scan, we reconstructed the size and location of the components of the conduction axis, using as explained the dimensional data available in the literature and their relative positions with respect to specific anatomical markers. We then proceeded to re-evaluate and, when necessary, confirm the location and extent of individual components based on our ability to record specific potentials and endocavitary electrograms (see Figure 4).

**Figure 5:**
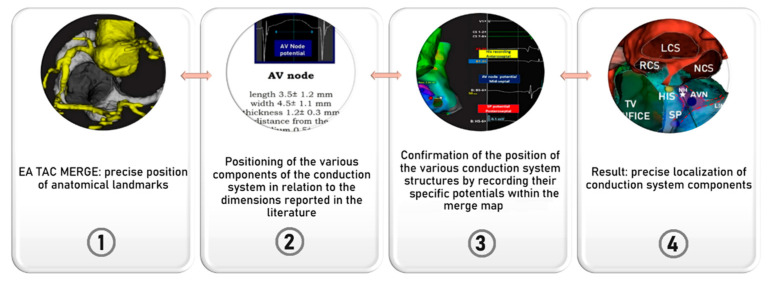
Flowchart of the proposed method to localize on the map the components of the atrioventricular conduction axis.

In all five patients, the near-field site of recording of the non-branching bundle devoid of any evidence of a nodal potential (His bundle) was identified at the site of the atrioventricular membranous septum. This coincided with the left-sided base of the interleaflet triangle between the non-coronary and right coronary sinuses. The low-frequency, low-amplitude humped nodal potential between the atrial and ventricular electrograms was consistently recorded within the presumed location of the compact node. This was positioned within the true septal area in 3 patients, or in the mid-paraseptal region in the other two patients. The average length of the region was 4.5 ± 1.2 mm, with its width measured at 2.7 ± 0.6 mm. The hump-like low-frequency, low-amplitude nodal potential, followed by a low-frequency His-like potential preceding the actual near-field His bundle potential was also recorded and may account for the recording of electrical activity at the site of compact AV node-His bundle transition, or may represent the potential of the lower nodal bundle. The hump like, low frequency potential was also recorded following the Jackmann slow pathway potential in the inferior para-septal region, superior to the orifice of the coronary sinus. The median distance from the slow pathway potential to the site of His potential was 15 ± 3.2 mm. This suggests that this low frequency potential recorded together with the Jackmann slow pathway potential likely represented the medial or distal portion of the inferior nodal extension, rather than the node itself. In Figure 3 and Figure 4, we provide an example of the merged acquisition, along with the corresponding methodology, while in Figure 5, we illustrate the flowchart used to pinpoint the components of the conduction axis. In all five patients, successful ablation was achieved, requiring a median of 5 [4–6] radiofrequency applications.

No procedural complications were observed. The effective ablation site, defined as the region where a junctional rhythm was elicited, followed by non-inducibility of the arrhythmia, was identified in the inferior para-septal area, bounded by the tricuspid annulus, in 3 patients, and in the mid para-septal region in 2 patients. In retrospect, we have observed that RF was not erogated within the region where the nodal potential was recorded without an accompanying slow pathway potential. All pulses were directed toward the anticipated site of the inferior nodal extension, where the hump-like, low-frequency, low-amplitude nodal potential was recorded in conjunction with the Jackmann slow pathway potential.

## 4. Discussion

For the first time, as far as we are aware, we have succeeded in correlating the presence of specific bipolar electrograms recordings with high-density electroanatomical mapping of the Koch’s triangle fused with imported high-quality anatomical computed tomographic scans. In this way, we have reliably and accurately localized the components of the atrioventricular conduction axis responsible for normal conduction and atrioventricular nodal re-entry tachycardia.

The area reconstructed, well described now as Koch’s pyramid [15], has a complex geometry, involving the wall of the right atrium, the underlying crest of the muscular ventricular septum, the pyramidal space and the infero-septal recess of the left ventricular outflow tract. The complexity is further compounded by the subendocardial location of the components of the conduction axis within the pyramid. From an anatomical standpoint, the Koch’s triangle is the right-side face of the pyramid; the base of the triangle is formed by the orifice of the coronary sinus and the septal isthmus, while the hinge of the septal leaflet of the tricuspid valve and the tendon of Todaro delineates its inferior and anterosuperior boundaries. The sides of the triangle meet at the apex, which is formed by the atrioventricular component of the membranous septum. This fibrous component is in turn confluent with the roof of the infero-septal recess, formed by an area between the aorto-mitral continuity and the interleaflet triangle between the non-coronary and right coronary aortic valve sinuses. A precise understanding of the location of the components of the atrioventricular conduction axis relative to these structures is of paramount importance for those undertaking ablations of arrhythmias arising in septal and para-septal areas and conduction system pacing. In consequence, numerous attempts have been made to correlate these aspects using histological studies. For example, Nagarajan and colleagues [16] measured the non-branching bundle with a length of 2.9 mm, a width of 7.3 mm, and a thickness of 1.1 mm, with a distance of 0.5 mm from the endocardial surface. Additionally, they reported the compact node to be 5 mm in length, 5 mm in width, and 1 mm in thickness. Others had previously reported similar measurements [9], while the right inferior nodal extension had been calculated at a length of 10.7 ± 1.3 mm, and measured at 8.2 ± 2.4 mm using histological samples [17]. More recently [18], the non-branching bundle has been measured with an average length of 3.6 mm. While histological studies are undoubtedly valuable, they cannot provide insights into the electroanatomic characteristics and activation patterns of the components of the atrioventricular conduction axis. Presently, furthermore, none of the available imaging techniques can accurately localize the components within the pyramid of Koch. This limits preoperative planning, and currently compromises procedural safety and efficacy. In this study, by seamlessly integrating electroanatomical mapping with reconstructed computed tomographic images, and taking into consideration the landmarks established by scans relative to the specific recorded electrograms, we present a proof of concept which demonstrates the feasibility of identifying precisely the locations of various anatomical components within the triangle (see Figure 4). The fusion of the electro-anatomic map with the reconstructed computed tomographic datasets, along with the concurrent analysis of the electrograms recorded in this region, has permitted us to define the position, length, and interrelationships of the boundaries and key anatomical components of the pyramid of Koch. Our estimations of the locations are endorsed by the recordings made at the site of ablation of the slow pathway. The persistent presence of the Jackmann slow pathway potential and a humped, low frequency electrogram at this site strongly suggests the anatomical presence of the right inferior nodal extension, while the absence of the compact node is indicated by the absence of its characteristic potential (hump-like, low frequency potential not preceded by a Jackmann slow pathway potential). The interpretation and annotation of these atypically prolonged electrograms rely fundamentally on foundational biophysical and physiological concepts derived from Sherlag’s pioneering studies in animal models [4]. This interpretation relies on foundational biophysical observations from Scherlag’s experimental studies and on the fact that, although ex vivo optical mapping of the human atrioventricular junction has been reported, direct transmembrane or intracellular recordings from the human AV node are not available in routine in vivo clinical electrophysiology [19]. A significant constraint of our approach lies in the absence of direct anatomical correlation in humans, due to the limited resolution of conventional imaging techniques, which do not permit precise visualization of the conduction system. Furthermore, the anatomical configuration of the canine heart—used in Sherlag’s investigations—differs from that of humans, particularly regarding the presence of the inferoseptal recess and the insulating structure surrounding the conduction axis.

Despite these limitations, the method employed to detect and analyze putative AV nodal electrograms represented the only viable strategy at the time, given the ethical and technical barriers to performing intracellular recordings and histological validation in living human subjects. Nonetheless, progress is being made in this field. Preclinical molecular imaging approaches have enabled in vivo visualization of the cardiac conduction system in animal models, supporting the feasibility of targeted identification of conduction tissue. In parallel, recent human anatomical and imaging studies have improved the indirect localization of the atrioventricular conduction axis by using reproducible cardiac CT landmarks and high-resolution hierarchical phase-contrast tomography. These advances may progressively allow a more accurate anatomical definition of the conduction system in relation to structures such as Koch’s triangle, the left ventricular outflow tract, and the aortic valve leaflets [20].

Currently, no clinical imaging modality offers the spatial resolution required to visualize the conduction tissue directly. However, it is possible to infer its location by referencing spatial relationships to adjacent cardiac structures visible via modalities such as cardiac CT [21]. This method serves as a bridge until advanced imaging tools, such as hierarchical phase-contrast tomography, which can achieve microscopic resolution, become feasible for clinical application [22]. Establishing a reproducible correlation between anatomical landmarks and specific intracardiac signals would mitigate concerns related to anatomical variability between species, as well as individual variation within the human conduction axis [23]. For instance, the positioning of the AV node within Koch’s triangle, the penetrating bundle, and the length of the non-branching bundle are all subject to anatomical variability. Although the Orion catheter was used, similar results may be achievable with other high-density mapping systems, provided adequate signal filtering is applied.

The precise delineation of the location of the conduction axis may clarify the pathways and mechanisms responsible for nodal re-entry tachycardia, which are currently but partially understood. Furthermore, knowledge of the location of the compact node, acquired through the recording of low-frequency and low-amplitude nodal potentials not preceded by a slow pathway potential has the potential to enhance the safety and efficacy of radiofrequency ablation procedures. Indeed, ablation for conditions such as ventricular ectopic beats, atrioventricular reentrant tachycardia, atrial tachycardia, and AV nodal re-entry tachycardia originating in the septal and paraseptal regions, would no longer be made in the sites where these electrograms are recorded. Conversely, these sites could serve as targets for the atrioventricular junction ablation specifically aimed at the compact node. Additionally, the accurate location of the non-branching and branching elements of the ventricular component of the conduction axis may facilitate the implantation of leads for pacing, thereby mitigating the risk of complications and reducing procedural and fluoroscopy durations. Ultimately, our research may pave the way for future investigations aimed at elucidating the specific roles of the components of the conduction axis found within the pyramid of Koch. This could establish a link between electrophysiological signatures and their anatomical substrates. Furthermore, such electrophysiological-anatomical correlations may yield potential targets for ablation of arrhythmias originating in the septal and paraseptal areas. We anticipate larger studies to validate and expand upon our current findings.

### Limitations

We recognize, nonetheless, that our study has several limitations. Firstly, our current cohort is small, thus restricting the generalizability of our findings. The agreement in measurements between the two methods across all five patients, nonetheless, demonstrates the satisfactory reproducibility of our methodology. It would be beneficial to explore additional cases, especially anatomical variations that may alter the relationships among the components of the atrioventricular conduction axis compared to the standard observed in our sample. The software utilized for segmentation of our computed tomographic datasets is open-source, and not specific to the mapping system employed. Some mapping systems employ their own proprietary software, which could potentially enhance integration. The open-source software, however, has been widely used and offers a blend of manual and semi-automatic tools to enhance user efficiency and facilitate a smooth learning curve. While operator-dependent variability in measurements is a potential concern, we mitigated this by having two independent technical specialists perform the measurements, resulting in nearly identical values. Finally, we acknowledge that the principles of biophysics and physiology we applied to interpret and annotate the humped, low-frequency, low-amplitude, and long-duration electrograms are rooted in the pioneering work of Sherlag and his colleagues [4,11]. We cannot directly record transmembrane action potentials from the atrioventricular node in humans. We have demonstrated, nonetheless, that by using unconventional bipolar filters, combined with high-density mapping catheters, it is possible to record potentials akin to those recorded and validated in the experimental studies. Further research is warranted to refine both the recording and proper annotation of the potentials recorded from the nodal structures and to enhance the quality of the images acquired through the various methods.

## 5. Conclusions

In conclusion, by combining three-dimensional electroanatomical maps with reconstructed computed tomographic datasets, and using specific anatomical landmarks, we propose a proof-of-concept method to estimate the location of components of the atrioventricular conduction axis relative to the boundaries of the pyramid of Koch. We submit that such accurate identification can enhance the safety and efficacy of transcatheter ablation in the septal and paraseptal regions, as well as the implantation of pacing leads.

## Data Availability

The data underlying this article will be shared on reasonable request to the corresponding author.
